# Associations between Nugent‐bacterial vaginosis and preterm birth and other adverse pregnancy outcomes in rural northwestern Bangladesh

**DOI:** 10.1002/ijgo.70691

**Published:** 2025-12-05

**Authors:** Daniel J. Erchick, Susan Tuddenham, Lena Kan, Lee S. F. Wu, Alain B. Labrique, Hasmot Ali, Ethan K. Gough, Mahbubur Rashid, Towfida J. Siddiqua, Subhra Chakraborty, Pawel Gajer, Michael France, Jacques Ravel, Golsa M. Yazdy, Keith P. West, Parul Christian

**Affiliations:** ^1^ Department of International Health Johns Hopkins Bloomberg School of Public Health Baltimore Maryland USA; ^2^ Division of Infectious Diseases Johns Hopkins School of Medicine Baltimore Maryland USA; ^3^ World Health Organization Geneva Switzerland; ^4^ JiVitA Project Rangpur Bangladesh; ^5^ Institute for Genome Sciences University of Maryland School of Medicine Baltimore Maryland USA; ^6^ Department of Gynecology and Obstetrics Johns Hopkins School of Medicine Baltimore Maryland USA

**Keywords:** bacterial vaginosis, Bangladesh, low birth weight, Nugent score, Preterm birth, small‐for‐gestational age, vaginal microbiota

## Abstract

**Objective:**

Bacterial vaginosis, defined by Nugent score (Nugent‐BV), has been associated with preterm birth and other adverse pregnancy outcomes. However, few studies have longitudinally described the associations between Nugent‐BV and adverse pregnancy outcomes at varying times in pregnancy.

**Methods:**

We assessed associations between Nugent‐BV at two separate time points in pregnancy and preterm birth (<37 weeks of gestation), low birth weight (LBW, <2500 g), and small‐for‐gestational‐age (SGA, <10th percentile) using data from a community‐based randomized trial in rural northwestern Bangladesh from 2001 to 2007. Pregnant women provided self‐collected vaginal swabs in early (first or second trimester) and late (third trimester) pregnancy for Nugent scoring. Nugent‐BV was categorized as 7–10 (ref: 0–6) or 4–10 (ref: 0–3). We used multivariable regression techniques to model relationships between Nugent‐BV and incidence of preterm birth, LBW, and SGA, adjusted for the trial intervention and demographic, socioeconomic, and pregnancy history factors.

**Results:**

A total of 1243 women provided ≥1 vaginal swab with a Nugent score and had a live birth. Preterm birth incidence was 23.5% (*n* = 271/1151). Nugent‐BV 7–10 in early (adjusted relative risk (aRR): 0.84, 95% confidence interval [CI]: 0.42, 1.66) and late (aRR: 1.04, 95% CI: 0.44, 2.41) pregnancy were not associated with preterm birth. Nugent‐BV 4–10 at either time point was not associated with preterm birth in the final adjusted models. From early to late pregnancy, a transition from Nugent score category 4–10 to 0–3, relative to 0–3 at both timepoints, was associated with decreased risk of preterm birth (aRR 0.20, 95% CI: 0.06, 0.63). In early and late pregnancy, neither Nugent‐BV categorization was associated with LBW or SGA in the final models.

**Conclusion:**

Resolution of Nugent‐BV between early and late pregnancy might be associated with a decreased risk of preterm birth. Longitudinal studies with frequent sampling in pregnancy and using molecular techniques are needed to better describe changes in the vaginal microbiota and associated risk for adverse pregnancy outcomes.

## 1 INTRODUCTION

In 2020, over half (55.3%) of the 2.4 million neonatal deaths globally were attributable to preterm birth (<37 weeks), small‐for‐gestational‐age (SGA), or their combination.^1^ Annually, approximately one in 10 live births (13.4 million) are preterm and one in five (23.4 million) are SGA, defined as <10th percentile of expected birthweight for gestational age and sex compared to a reference standard.^2^ Rates of preterm birth and other adverse pregnancy outcomes have largely remained unchanged over the past decade.^1^, ^2^ The highest rates of preterm birth and SGA occur in low‐ and middle‐income countries (LMICs), particularly South Asia, where few interventions exist to prevent these outcomes and therapeutic interventions to improve survival are often unavailable.^3^

Preterm birth is a complex, multifactorial condition with many risk factors, but a large proportion of spontaneous preterm births have been attributed to infection and inflammation, particularly intrauterine infections ascending from the vagina.^4^ Bacterial vaginosis (BV), a dysbiosis of the vaginal microbiota, whether symptomatic or asymptomatic, has been associated with adverse pregnancy outcomes, including preterm birth, miscarriage, and stillbirth.^5^, ^6^ BV is characterized by a decrease in beneficial *Lactobacillus* species and increase in a diverse composition of anaerobic species, including *Gardnerella*, *Prevotella*, *and Mobiluncus species*, in the vagina.^7^

Bacterial vaginosis can be diagnosed in several ways, but the Nugent score has been commonly used to examine relationships between the vaginal microbiota and adverse pregnancy outcomes.^7^ This method uses a gram‐stained vaginal smear to determine relative concentrations of three bacterial morphotypes (*Lactobacillus*, *Gardnerella/Bacteroides*, *and Mobiluncus* species).^7^ Scores are defined as 0–3 for an “optimal” or “low‐risk” *Lactobacillus*‐dominated vaginal community and 7–10 for BV, characterized by a decrease in *Lactobacillus* and increase in Nugent‐BV‐associated taxa.^7^ A score of 4–6 is considered “intermediate” and 4–10 is often classified as abnormal vaginal microbiota.^8^

Nugent‐BV 7–10 has been linked with preterm birth in observational studies across populations.^6^, ^9^ A meta‐analysis of 18 studies found a twofold increase in risk of preterm delivery among women with Nugent‐BV 7–10 (odds ratio [OR]: 2.19 95% confidence interval [CI]: 1.54, 3.12).^9^ Fewer studies have assessed associations between Nugent‐BV 4–6 or 4–10 categorizations and preterm birth.^8^ Another meta‐analysis reported that Nugent‐BV 4–6 was not associated with preterm delivery in a sub‐group of 5 studies; although several studies showed positive trends but were inadequately powered to examine this question due to small sample sizes.^8^ One study in rural Bangladesh reported that women between 13 and 19 weeks of gestation with Nugent‐BV 4–10 who were treated for BV but not cured had higher risk (RR: 1.33, 95% CI: 1.07, 1.65) of adverse pregnancy outcomes, relative to those who did not have Nugent‐BV 4–10.^10^

Screening and treatment of Nugent‐BV in pregnancy, most commonly with antibiotics such as metronidazole or clindamycin, has not consistently led to improvements in preterm birth or other adverse pregnancy outcomes.^11^, ^12^, ^13^, ^14^, ^15^ One systematic review and individual participant data analysis from multiple trials showed no effect for either antibiotic (metronidazole: OR: 0.95, 95% CI: 0.81, 1.11; clindamycin: OR: 0.90, 95% CI: 0.72, 1.12).^11^ A population‐based screening and treatment program for Nugent‐BV 4–10 and urinary tract infections in rural Bangladesh did not impact preterm birth, and authors highlighted several challenges facing intervention trials of this association, including achieving high effective treatment coverage, particularly with variation in microbial composition that is characteristic of Nugent‐BV 4–6.^16^ New treatments for vaginal dysbiosis are under development, and randomized trials will be needed to determine whether such interventions could prevent preterm birth and other adverse pregnancy outcomes.^17^

Women living in Bangladesh are significantly impacted by Nugent‐BV and high rates of preterm birth and other adverse pregnancy outcomes.^10^, ^18^ From 2001 to 2007, the JiVitA research team conducted a double‐blind, cluster‐randomized, placebo‐controlled trial of weekly maternal vitamin A and β‐carotene supplementation, within which was nested a sub‐study to measure Nugent‐BV prevalence in early and late pregnancy and postpartum in rural northwestern Bangladesh.^19^, ^20^, ^21^ The study reported that neither the prevalence nor incidence of Nugent‐BV 7–10 differed by supplement group in the third trimester. However, vitamin A supplementation reduced the prevalence (OR: 0.71; 95% CI: 0.52, 0.98) and incidence (RR: 0.58; 95% CI: 0.41, 0.81) of Nugent‐BV 7–10 at 3 months postpartum compared to placebo, and both vitamin A and β‐carotene reduced the prevalence and incidence of Nugent‐BV 7–10 at either late pregnancy or postpartum by 30–40% compared to placebo.^20^ A recent secondary analysis of these data identified participant factors associated with increased risk of Nugent‐BV in early and late pregnancy (including older age, Hindu religion, and shorter pregnancy interval) and factors associated with decreased risk (including always bathing with soap, higher socioeconomic status, receiving more antenatal care visits, and lower body mass index [BMI]).^18^

Using data from this large randomized controlled trial conducted in rural northwestern Bangladesh, we examined the associations between Nugent‐BV 7–10 and 4–10 in early and late pregnancy and the incidence of preterm birth and other adverse pregnancy outcomes.

## 2 METHODS

### 2.1 Data collection

Data for this analysis were collected through the JiVitA‐1 study, a double‐blind, cluster‐randomized, placebo‐controlled trial of maternal vitamin A and β‐carotene supplementation conducted from 2001 to 2007 in the rural northwestern districts of Gaibandha and Rangpur in Bangladesh.^19^ The study enrolled approximately 60 000 pregnant women over an area of approximately 450 km^2^ divided into 596 communities called sectors, which were used as the unit of randomization for supplement allocation. Participants for this study were recruited between September 9, 2002, and December 26, 2006, and randomly assigned to one of the three trial arms. Vitamin A was provided as a directly observed weekly dose in a gelatin supplement at 7000 µg retinol equivalents (REs) in the form of retinyl palmitate and β‐carotene at 42 mg, equivalent to 7000 µg REs.^21^, ^22^ All three supplements, including the placebo, contained 5 IU vitamin E in oil and were identical in appearance. Neither supplement reduced the primary outcome of this trial of pregnancy‐related mortality nor secondary outcomes of gestational age at birth, preterm birth, low birth weight, SGA, and birth weight, length, and chest, head, and arm circumferences.^19^, ^23^

The BV sub‐study, which is the focus of this secondary analysis, was conducted in 32 sectors of the JiVitA‐1 study area in Jamalpur Union, Gaibandha District. The sub‐study area covered a population of approximately 30 000 people, including 6000 women of reproductive age. All women of reproductive age in the community were visited every 5 weeks by local female data collectors to ask if participants had a period since the last visit, offer a urine‐based pregnancy test if not, and record the date of the first day of the last menstrual period (LMP), an approach validated to have high sensitivity and specificity.^24^ All women identified as pregnant were enrolled in the trial by experienced female interviewers and visited for interviews at baseline, in the third trimester, and at 3‐ and 6‐months postpartum. Interviewers collected data on participant demographic factors, socioeconomic status, pregnancy history, diet and nutrition, pregnancy morbidities, labor and delivery characteristics, and maternal and infant anthropometry. A cadre of local female project staff visited all trial participants weekly to provide the study supplement, assess compliance, record the vital status of women and infants, and report pregnancy outcomes. The sub‐study was established to include an additional set of nutritional and anthropometric assessments conducted at participant homes in early pregnancy (first or second trimester), in late pregnancy (third trimester), and 3 months postpartum, including a questionnaire on personal hygiene, reproductive symptoms, and sexual behaviors and collection of self‐administered vaginal swabs for Nugent scoring and diagnosis of BV. Early visits were defined as those that occurred before 28 weeks' gestation and late visits those from 28 weeks' gestation to delivery. Vaginal swabs were collected from women at each time point regardless of whether swabs had been collected at previous visits to maximize the number of swabs collected at each cross‐section. At any time point, women with a gram stain score 7–10 and self‐report of abnormal vaginal discharge were treated with a 7‐day regimen of metronidazole (250 mg, three times a day).

The objective of this secondary analysis was to assess the associations between Nugent‐BV and preterm birth and adverse pregnancy outcomes, adjusting for trial nutritional supplementation and participant demographic, socioeconomic, and behavioral factors, using population‐based data from rural northwestern Bangladesh (Table S1).

The JiVitA‐1 study received ethical approval for all study procedures, including the verbal consent process, from the Johns Hopkins School of Public Health Institutional Review Board in Baltimore, Maryland, United States, and the Bangladesh Medical Research Council in Dhaka, Bangladesh. All participants provided verbal informed consent. JiVitA data collectors visited participants in their homes to obtain and document verbal consent for study participation. The trial is registered at clinicaltrials.gov (NCT00198822). The principal investigator of the JiVitA‐1 trial (KPW) authorized use of the trial data for this secondary analysis.

### 2.2 Nugent‐bacterial vaginosis definitions

At each study visit, vaginal swabs (Dacron Polyester‐tipped sterile applicator swabs, Puritan Medical Products, Guildford, ME) were collected by participants through insertion into the vagina, approximately 2 inches, and gently rubbing the lateral wall for a few seconds. The swabs were then passed from the participant to the technician for smearing onto a glass microscope slide for Gram staining and Nugent scoring. Technicians used an electric binocular microscope to assess 10 fields on each slide, assigning for each a summed numerical score from 0 to 10 based on morphology and Gram stain of vaginal organisms, including *Lactobacillus* (decreasing score from 4 to 0), *Gardnerella vaginalis* (increasing score from 0 to 4), and *Mobiluncus* (increasing score from 0 to 2).^7^ A sample (7.5%) of slides were assessed again by a study co‐investigator (ABL) for quality control. Discordant results were discussed with the technicians until a consensus on a final score was reached. For analysis, we used the median Nugent of 10 fields to categorize each swab as Nugent‐BV 7–10 or 4–10, intermediate Nugent‐BV 4–6, and no Nugent‐BV 0–3. We also constructed categorical variables to describe Nugent score category transition from early to late pregnancy as 0–3 both time points, 4–10 early to 0–3 late, 0–3 early to 4–10 late, or 4–10 both time points.

### 2.3 Adverse pregnancy outcomes definitions

Our outcome of interest for this analysis was preterm birth defined as gestational age at delivery <37 weeks among live births.^25^ Gestational age was calculated as the difference in days between the date of pregnancy outcome and the LMP date as recalled by the mother at enrollment early in pregnancy, a dependable marker of gestational age in this community.^23^ We defined several other pregnancy outcomes at delivery among live births, including binary classifications of LBW as <2500 g and SGA as less than the 10th centile for exact gestational age in days by sex using the INTERGROWTH‐21st Standard.^26^ For outcomes requiring birth weight, we excluded weights collected >72 h from delivery.

### 2.4 Statistical analysis

This secondary analysis used data from participants enrolled in the JiVitA‐1 BV sub‐study, including those randomized across the three trial groups. Analyses were conducted separately for Nugent‐BV 7–10 and 4–10 in early pregnancy and late pregnancy and also considering transitions between Nugent 0–3 and 4–10 category from early to late pregnancy. We assessed bivariate associations between participant factors and Nugent‐BV categories and preterm birth. We calculated the incidence of preterm birth and other adverse pregnancy outcomes overall and by Nugent‐BV categories and assessed significance in bivariate analyses using Pearson's χ^2^‐test. We used multivariable Poisson regression models to estimate unadjusted and adjusted relative risks of preterm birth for Nugent 7–10 and 4–10 and associated 95% confidence intervals adjusting for participant characteristics. For LBW and SGA outcomes, we used a similar modeling approach. In all models, to account for the trial's cluster design, we adjusted the standard errors using generalized estimating equations with an exchangeable correlation structure. Multivariable adjusted models included covariates for trial supplement group, maternal age, parity, BMI, education, religion, living standard index, treatment for symptomatic BV, and exact gestational age at time of Nugent score measurement. These variables were selected a priori as potential confounders for the association between Nugent‐BV and preterm birth. We excluded multiple pregnancies from these analyses due to their high risk of preterm birth. Sensitivity analyses conducted by including additional variables of interest in final regression models did not result in substantial changes in the relationships between Nugent‐BV in early or late pregnancy and preterm birth. These variables included age at marriage, previous stillbirth or miscarriage, short pregnancy interval (<18 months, ≥18 months), water source for bathing (improved well, natural source), and type of family planning method used prior to pregnancy (non‐hormonal, estrogen/progestin, progestin only). Analyses were conducted using Stata 18.0 (Stata Corp, TX, USA).

## 3 RESULTS

**FIGURE 1:**
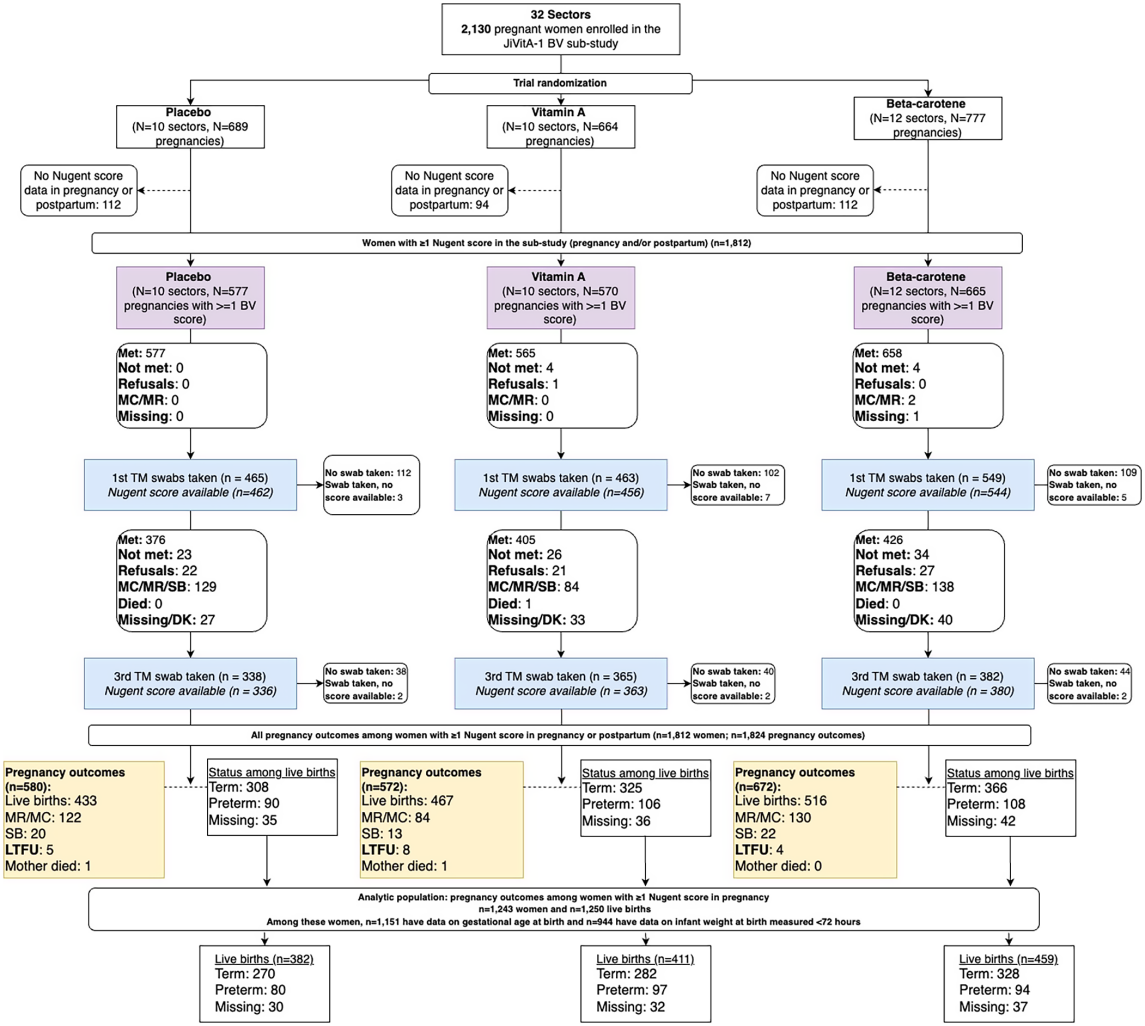
Participant flowchart for JiVitA‐1 BV sub‐study and analytic population for this analysis.

Among 2130 pregnant women enrolled in this sub‐study, 1628 (76.4%) had at least one vaginal swab that yielded a Nugent score in pregnancy (Figure 1). We restricted our analysis to the 1243 women who had at least one live birth and provided at least one swab and associated Nugent score in pregnancy. Of their 1250 infants, all had data on sex (100%), 1151 (92.1%) had data on gestational age at birth, and 944 (75.5%) had data on infant weight within <72 h of delivery. Of the 1243 women with a live birth and at least one swab, 1064 (85.6%) and 1038 (83.5%) had Nugent score data in early and late pregnancy, respectively. A total of 1136 singleton live births had data on our outcome of interest, preterm birth, and among these 970, 971, and 805 had Nugent score data at early pregnancy, late pregnancy, or both time points, respectively, for our final regression analyses.

The mean age (standard deviation [SD]) of participants was 21.0 (5.5) years and 661 (53.3%) were nulliparous (Table 1). Most participants were Muslim (*n =* 1162, 93.6%), 820 (66.1%) had some education, and 661 (53.2%) were literate. The prevalence of underweight was 41.7% (*n =* 516) and maternal short stature was 55.8% (*n =* 694) among women in early pregnancy. Twenty‐six (2.1%) women delivered via C‐section. Over three‐quarters (*n =* 838, 77.5%) of women at baseline reported sourcing their water for bathing in a pond, lake, or river (Table S2). Prior to pregnancy, one‐third of women (*n =* 348, 32.2%) reported using oral contraceptive pills and less than 10% (*n =* 91, 8.4%) used Norplant or Depo‐Provera. Nearly all women reported regularly having sex during early and late pregnancy (early pregnancy: *n =* 1073, 99.4%).

**TABLE 1:** Participant characteristics at baseline among pregnant women in rural Bangladesh.

	*n =* 1243
Supplement group^a^	
Placebo	378 (30.4%)
Vitamin A	409 (32.9%)
Beta carotene	456 (36.7%)
Gestational age at enrollment (weeks)	
<8	218 (18.2%)
8–<12	504 (42.2%)
≥12	473 (39.6%)
Gestational age at early pregnancy vaginal swabs	
Mean (SD)	11.9 (4.9)
Gestational age at late pregnancy vaginal swabs	
Mean (SD)	32.8 (2.0)
Age at enrollment (years)	
<18	361 (29.1%)
18–29	769 (62.0%)
≥ 30	111 (8.9%)
Age at first marriage (years)	
<15	565 (55.7%)
15–18	294 (29.0%)
≥ 18	156 (15.4%)
Parity	
0	661 (53.3%)
1	535 (43.1%)
≥ 2	45 (3.6%)
Previous stillbirth or miscarriage	
No	512 (83.9%)
Yes	98 (16.1%)
Pregnancy interval (months)	
≥ 18	522 (91.1%)
<18	51 (8.9%)
Maternal short stature (cm)	
≥ 150	549 (44.2%)
<150	694 (55.8%)
Body mass index	
Underweight	516 (41.7%)
Normal weight	693 (56.0%)
Overweight or obese	29 (2.3%)
MUAC (cm)	
<20	54 (4.4%)
≥ 20–<23	613 (49.5%)
≥ 23	571 (46.1%)
Religion	
Muslim	1162 (93.6%)
Hindu	80 (6.4%)
Education	
No schooling	421 (33.9%)
Class 1–7	455 (36.7%)
Class 8–14	365 (29.4%)
Literacy	
No	581 (46.8%)
Yes	661 (53.2%)
Living standard index	
− 0.20859	505 (40.7%)
≥ −0.20859	737 (59.3%)
Paid employment	
No	752 (60.5%)
Yes	490 (39.5%)
Grameen Bank membership	
No	1142 (91.9%)
Yes	100 (8.1%)
BRAC membership	
No	1082 (87.1%)
Yes	160 (12.9%)
House has electricity	
No	1064 (85.7%)
Yes	178 (14.3%)
Television ownership	
No	1106 (89.0%)
Yes	136 (11.0%)
Toilet type	
None/field/bush	498 (40.1%)
Open/hanging latrine	19 (1.5%)
Pit latrine	135 (10.9%)
Water sealed/slab	588 (47.3%)
Flush toilet	2 (0.2%)
Tobacco	
No	1142 (92.2%)
Yes	96 (7.8%)
Alcohol	
No	1238 (100.0%)
Yes	0 (0.0%)
Betel nut	
No	393 (32.0%)
Yes	834 (68.0%)
Multiple pregnancy	
Singleton	1235 (99.4%)
Multiple	8 (0.6%)

^a^ Number (%).

**FIGURE 2:**
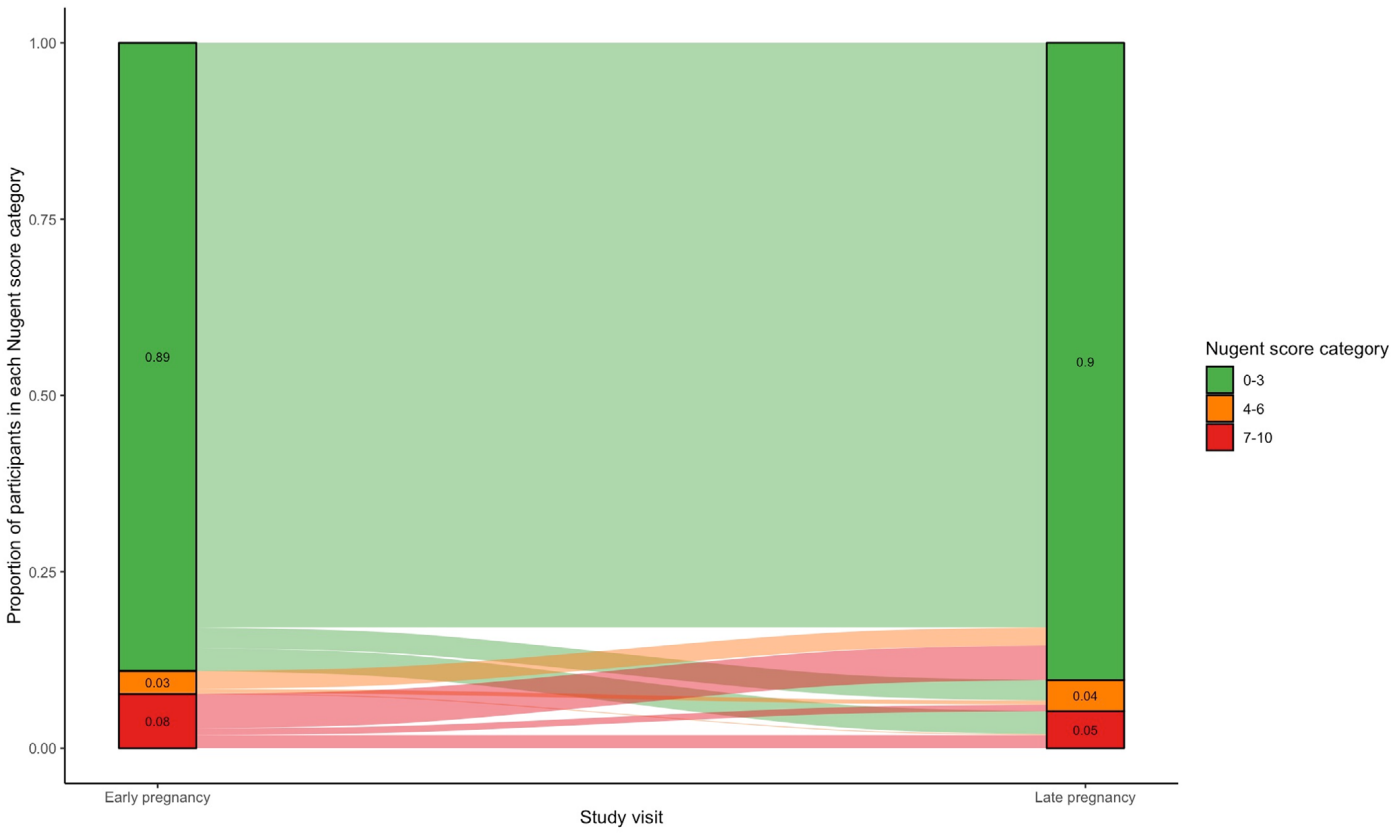
Nugent score category transitions from early to late pregnancy.^1^ This figure depicts participant Nugent score category status, including missing data, at the early and late pregnancy visit for *n =* 1243 women. Not recorded includes missing data for several reasons, including that participants missed the visit, participants declined to provide the swab, or a slide could not be read due to damage or other preparation error. In early pregnancy, these include *n =* 945 (88.8%) with 0–3, *n =* 34 (3.2%) with 4–6, and *n =* 85 (8.0%) with 7–10 (at this timepoint and ignoring missingness). In late pregnancy, these include *n =* 914 (88.1%) with 0–3, *n =* 56 (5.4%) with 4–6, and *n =* 68 (6.6%) with 7–10 (at this timepoint and ignoring missingness).

In early pregnancy, the prevalence of Nugent‐BV 7–10 was 8.0% (*n =* 85) and 4–10 was 11.2% (*n =* 119). In late pregnancy, the prevalence of Nugent‐BV 7–10 was 6.6% (*n =* 68) and 4–10 was 12.0% (*n =* 124). The majority of participants remained in the same Nugent score category between early and late pregnancy; 88.9% (*n =* 764) had Nugent 0–6 at both time points and 82.9% (*n =* 712) had Nugent 0–3 at both time points, while 1.9% (*n =* 16) had Nugent‐BV 7–10 at both time points and 3.5% (*n =* 30) had Nugent‐BV 4–10 at both time points (Figure 2). Fewer participants transitioned from Nugent‐BV 7–10 to Nugent 0–6 (*n =* 50, 5.8%) or Nugent 0–6 to Nugent‐BV 7–10 (*n =* 29, 3.4%). Similarly, few participants transitioned from Nugent‐BV 4–10 to Nugent 0–3 (*n =* 64, 7.5%) or Nugent 0–3 to Nugent‐BV 4–10 (*n =* 53, 6.2%).

In early and late pregnancy, the percentage of women with Nugent‐BV 7–10 who were symptomatic (self‐report of abnormal vaginal discharge) was 61.9% (*n =* 52/84) and 61.1% (*n =* 33/54), respectively. The majority of these women received treatment in early (*n =* 37/52, 71.2%) or late (*n =* 27/33, 81.8%) pregnancy (Table S3).

Overall, the incidence of preterm birth was 23.5% (*n =* 271/1151). These included 69.4% late (*n =* 188/273), 19.6% moderate (*n =* 53/271), and 11.1% very (*n =* 30/271) preterm births. Mean (SD) gestational age at birth was 38.6 (2.8) weeks. The incidence of SGA was 60.6% (*n =* 532/878) and LBW was 50.5% (*n =* 477/944). Mean (SD) birth weight was 2563 (650) grams.

For Nugent‐BV 7–10, there were no significant associations with preterm birth in unadjusted or adjusted models either in early (aRR: 0.84, 95% CI: 0.42, 1.66) or late (aRR: 1.04, 95% CI: 0.44, 2.41) pregnancy (Table 2). For Nugent‐BV 4–10, the association with preterm birth was similar in magnitude to that of Nugent‐BV 7–10 in early pregnancy (aRR: 0.84, 95% CI: 0.53, 1.33). However, in late pregnancy, Nugent‐BV 4–10 was borderline significantly associated with preterm birth in the unadjusted model (RR: 1.29, 95% CI: 0.95, 1.73), which was attenuated and not statistically significant in the final adjusted model (aRR: 1.20, 95% CI: 0.81, 1.77).

**TABLE 2:** Associations between Nugent‐BV 7–10 and 4–10 in early and late pregnancy and preterm birth.

Assessment time point*, ^+^	Nugent‐BV 7–10		Nugent‐BV 4–10	
	0–6	7–10	0–3	4–10
Early pregnancy				
Term^a^	690 (77.0%)	61 (82.4%)	667 (76.9%)	84 (81.6%)
Preterm	206 (23.0%)	13 (17.6%)	200 (23.1%)	19 (18.5%)
Unadjusted^b^	Ref	0.75 (0.48, 1.17)	Reference	0.79 (0.55, 1.13)
Adjusted	Ref	0.84 (0.42, 1.66)	Reference	0.84 (0.53, 1.33)
Late pregnancy				
Term	738 (81.5%)	51 (78.5%)	698 (81.8%)	91 (77.1%)
Preterm	168 (18.5%)	14 (21.5%)	155 (18.2%)	27 (22.9%)
Unadjusted	Ref	1.22 (0.67, 2.20)	Reference	1.29 (0.95, 1.73)^+^
Adjusted	Ref	1.04 (0.44, 2.41)	Reference	1.20 (0.81, 1.77)

^a^ Number (%).

^b^ Model results represent the unadjusted and adjusted relative risks of preterm birth for each time point and exposure category and 95% confidence intervals from Poisson regression models utilizing generalized estimating equations to account for the trial's cluster design. Adjusted models included covariates for trial supplementation group, maternal age, parity, body mass index, education, religion, living standard index, treatment of symptomatic bacterial vaginosis (BV) with antibiotics, and gestational age at time of Nugent score measurement (early or late pregnancy). Multiple births were dropped from regression models due to convergence issues. Models assessing the Nugent‐BV 7–10 exposure used Nugent score 0–6 as the reference group, while models assessing the Nugent‐BV 4–10 exposure used Nugent score 0–3 as the reference group. Models for early and late pregnancy time points were conducted separately for each exposure as independent analyses.

* *P* < 0.05.

^+^ *P* < 0.10.

Women who transitioned from Nugent 4–10 to 0–3 between early to late pregnancy had a lower risk of preterm birth (aRR: 0.20, 95% CI: 0.06, 0.63) relative to those with 0–3 at both time points (Table 3). Women who transitioned from Nugent 0–3 to 4–10 or had Nugent 4–10 at both time points did not have a statistically significantly different risk of preterm birth than those with Nugent 0–3 at both time points.

**TABLE 3:** Associations between Nugent‐BV transitions from early to late pregnancy and preterm birth.

Nugent‐BV category from early to late pregnancy*, +	Term birth^a^	Preterm birth	Relative risk (RR) (95% CI)^b^	aRR (95% CI)
0–3 both time points	547 (82.6)	122 (85.3)	Ref	Ref
4–10 early to 0–3 late	54 (8.2)	3 (2.1)	**0.29 (0.10, 0.86)** *	**0.20 (0.06, 0.63)** *
0–3 early to 4–10 late	39 (5.9)	11 (7.7)	1.25 (0.74, 2.12)	0.99 (0.49, 1.97)
4–10 both time points	22 (3.3)	7 (4.9)	1.29 (0.71, 2.32)	0.98 (0.50, 1.91)

Abbreviation: BV, bacterial vaginosis.

^a^ Number (%).

^b^ Model results represent the unadjusted and adjusted relative risks of preterm birth for each transition category and 95% confidence intervals (CIs) from Poisson regression models utilizing generalized estimating equations to account for the trial's cluster design. Adjusted models included covariates for the trial supplementation group, maternal age, parity, body mass index, education, religion, living standard index, and treatment of symptomatic BV with antibiotics. Multiple births were dropped from regression models due to convergence issues.

* Significant bold values *P* < 0.05.

^+^ *P* < 0.10.

In bivariate analyses, the proportion of SGA births was higher among women with Nugent 7–10 (*P* < 0.10) (Table S4). However, in final adjusted regression models, neither Nugent‐BV exposure category was associated with incidences of SGA or LBW births (Table 4, Table S5).

**TABLE 4:** Associations between Nugent‐BV 7–10 and 4–10 in early and late pregnancy and LBW and SGA.

Assessment time point*, +	SGA	LBW
Nugent‐BV 7–10^a^		
Early pregnancy	1.09 (0.88, 1.35)	0.97 (0.64, 1.46)
Late pregnancy	1.04 (0.78, 1.39)	1.08 (0.78, 1.52)
Nugent‐BV 4–10		
Early pregnancy	1.01 (0.84, 1.21)	0.87 (0.62, 1.22)
Late pregnancy	1.04 (0.85, 1.26)	1.08 (0.86, 1.36)
Nugent‐BV status early to late pregnancy^b^		
0–3 both time points	Reference	Reference
4–10 early to 0–3 late	1.14 (0.94, 1.38)	0.70 (0.46, 1.07)
0–3 early to 4–10 late	0.94 (0.71, 1.23)	0.95 (0.70, 1.29)
4–10 both time points	1.10 (0.86, 1.42)	1.16 (0.81, 1.66)

Abbreviations: LBW, low birth weight; SGA, small‐for‐gestational‐age.

^a^ Model results represent the adjusted relative risks of each adverse pregnancy outcome for each time point and exposure category and 95% confidence intervals from Poisson regression models utilizing generalized estimating equations to account for the trial's cluster design.

^b^ Model results represent the adjusted relative risks of each adverse pregnancy outcome and transition category and 95% CIs from Poisson regression models utilizing generalized estimating equations to account for the trial's cluster design. Adjusted models included covariates for trial supplementation group, maternal age, parity, body mass index, education, religion, living standard index, and treatment of symptomatic bacterial vaginosis (BV) with antibiotics. Multiple births were dropped from regression models due to convergence issues.

* *P* < 0.05.

^+^ *P* < 0.10.

## 4 DISCUSSION

### 4.1 Main findings

In rural northwestern Bangladesh, we found that women with Nugent‐BV 7–10 in early or late pregnancy were not at increased risk of preterm birth. In late pregnancy, Nugent‐BV 4–10 was borderline significantly associated with preterm birth in the unadjusted model but not statistically significant in the final adjusted model. Women who transitioned from Nugent 4–10 to 0–3 between early and late pregnancy, compared to those in 0–3 at both timepoints, had a significantly lower risk of preterm birth. These findings suggest that risk for preterm birth associated with Nugent‐BV might vary by when the exposure is present during the course of pregnancy. The finding of lower risk among those who transition to a state absent of Nugent‐BV later in pregnancy suggests the possibility that this exposure could influence proximal pathways for the initiation of early spontaneous delivery.

### 4.2 Interpretation

The prevalence of Nugent‐BV 7–10 in our population decreased slightly from early to late pregnancy (8.0% to 6.6%), while Nugent 4–10 remained roughly the same (11.2% to 12.0%). The prevalence of Nugent‐BV 7–10 in our study was similar (6.8%) to a previous study reporting on a population of pregnant women in Sylhet District in northeastern Bangladesh who were assessed between 13 and 19 weeks of gestation.^10^ However, in the Sylhet population, the prevalence of Nugent score 4–6 was higher (9.8%) than in our study in northwestern Bangladesh, where we observed an increase from 3.2% to 5.4% between early and late pregnancy.^10^ A meta‐analysis of global BV prevalence reported a regional prevalence of 11.7% for South Asia (95% CI: 9.0, 14.7), which was lower than other regions, including 29.1% in Sub‐Saharan Africa and 33.2% in Latin America and the Caribbean.^27^ Outside pregnancy and especially postpartum, prevalence of BV is typically higher; in a recent review, the prevalence of BV in the general population was 28.7% for the South Asia region (95% CI: 21.2, 36.8).^27^

Studies of the BV and preterm birth relationship have used varying exposure definitions and assessment time points in pregnancy.^6^, ^8^ A strength of our study was the opportunity to assess Nugent‐BV exposures both in the first and third trimester on risk of preterm birth and other adverse pregnancy outcomes in a population with high risk for these conditions and low levels of important confounders, such as chronic diseases.^23^ The study in Sylhet District, Bangladesh, showed that women with persistent Nugent score 4–10 (defined as presence of Nugent 4–10 3 weeks after BV treatment) in late first and second trimester had higher risk (aRR: 1.33, 95% CI: 1.07, 1.65) of a composite outcome, including preterm live birth, preterm stillbirth, and miscarriage.^10^ A study by Donders et al. (2009), reported that women without Nugent‐BV (0–3), relative to those with Nugent‐BV 4–10, had 75% lower odds of preterm birth (OR: 0.26, 95% CI: 0.12, 0.56)^28^ in addition to lower risk of miscarriage. These authors distinguished “full BV” from “partial BV,” which they defined as patchy streaks of BV flora or sporadic clue cells mixed with other flora, finding that partial BV was significantly associated with preterm birth, while full BV was not.^28^ One review, which looked specifically at intermediate Nugent scores (Nugent‐BV 4–6) and risk of preterm birth and other adverse outcomes, found no significant relationships, although authors noted positive trends and suggested that their non‐significant results might be due to small sample size or heterogeneity in the vaginal microbiota of patients with intermediate flora.^8^

Although meta‐analyses have consistently showed that antibiotic treatment for BV has failed to reduce risk of preterm birth, a Cochrane review reported some evidence that treatment of Nugent‐BV 4–10 might reduce risk of preterm birth (RR: 0.53, 95% CI: 0.34 to 0.84), although this sub‐group analysis only included two trials and should, therefore, be interpreted with caution.^12^ In our study, it is possible that an association between Nugent‐BV and preterm birth was attenuated by treatment of symptomatic BV+ women with antibiotics, especially for those treated early in pregnancy.

Meta‐analyses have reported conflicting results about how the timing of Nugent‐BV exposure in pregnancy affects the subsequent risk of preterm birth. One meta‐analysis, by Leitich et al. (2003), initially showed that women screened at <20 and <16 weeks had a higher risk of preterm birth; however, in an updated review, they contradicted this finding, reporting that early pregnancy Nugent‐BV presented no additional risk beyond the overall association.^8^, ^9^ In contrast, a more recent meta‐analysis of 22 studies found that risk for maternal and fetal outcomes was significantly higher among women who tested positive for Nugent‐BV in the third trimester.^6^ Our findings support a hypothesis that transition from a high to low‐risk state over the course of pregnancy might be protective for preterm birth.

Many factors in pregnancy, including age, blood pressure, smoking, infection, anemia, undernutrition, are associated with fetal growth restriction and increased risk SGA and LBW birth.^29^, ^30^, ^31^, ^32^ However, in our study, we did not find significant associations between Nugent‐BV and LBW or SGA. A recent meta‐analysis of maternal and fetal outcomes found a significant association between Nugent‐BV 7–10 and LBW (OR 1.73, 95% CI 1.41, 2.12).^6^ Further research should consider associations between the vaginal microbiota and detailed phenotypes of vulnerable newborns, defined by SGA, preterm, and LBW.^33^, ^34^, ^35^

### 4.3 Strengths and limitations

This study had several strengths and some limitations. The community‐based cohort design enrolling women early in pregnancy and assessment of Nugent score at two time points allowed us to prospectively observe this relationship and reduce risk of selection bias associated with other designs (e.g., case–control) and settings (e.g., facility‐based) that are common among previous studies of this relationship. However, we were not able to collect data on some key variables that could represent important confounders of the BV and preterm birth relationship, including participant sexual history and previous preterm birth. The absence of a clinical exam and reliance on self‐collected vaginal swabs and self‐reported symptoms of abnormal vaginal discharge and other symptoms present risk of measurement and reporting biases in our primary exposure. Not all symptomatic BV+ participants in our study received treatment for BV, treatment adherence was not high, and there was no clinical follow‐up to determine treatment success or failure, which would have been an important factor to control for in our final analysis. Although our analytical sample had a large number of preterm births overall, the numbers of preterm births among those with Nugent‐BV at early or late pregnancy cross‐sections or transition categories were small, which can result in unstable risk estimates, wider confidence intervals, and an increased influence of misclassification on the results. Our primary outcome of preterm birth was based upon the LMP method, which although known to be less accurate than ultrasonography, has been shown as a reliable indicator of gestational age at delivery in this community.^24^ Lastly, although long considered the gold standard for diagnosis of bacterial vaginosis, Nugent scoring is a non‐specific classification that masks complex and rapidly changing microbial compositions and immunologic and metabolomic activity in the vaginal environment.

## 5 CONCLUSION

Our findings support the need for continued research to understand the relationship between Nugent‐BV and preterm birth and other adverse pregnancy outcomes. Longitudinal studies with frequent sampling in pregnancy, and even spanning preconception to postpartum, might be required to determine the time of highest risk. This is particularly important given the heterogeneity in risk by gestational age at Nugent‐BV screening observed in previous studies, the rapidly changing nature of the vaginal microbiota, and the potential influence of short‐term shifts to higher risk compositions.^36^ New molecular techniques to describe vaginal microbial compositions and specific taxa, in the context of host inflammatory responses, will provide a more detailed description of the microbial exposures and vaginal environmental conditions that associate with an increased or decreased risk for adverse pregnancy outcomes. Ultimately, randomized trials will be needed to evaluate new interventions for their ability to treat Nugent‐BV and dysbiosis of the vaginal microbiota and promote the long‐term maintenance of a vaginal environment with low‐risk *Lactobacillus crispatus* species to reduce risk for adverse pregnancy outcomes.

## AUTHOR CONTRIBUTIONS

DJE, ST, ABL, EG, SC, and JR contributed to the study concept and design. DJE conducted the analysis and wrote the manuscript. ABL and DJE obtained funding for this secondary analysis. KPW obtained funding for the parent study. All authors reviewed results, discussed interpretations, and contributed to development and revision of the manuscript.

## FUNDING INFORMATION

This work was supported by the Bill and Melinda Gates Foundation as part of Grant OPP1211544. The original research study was funded by the Bill and Melinda Gates Foundation (Global Control of Micronutrient Deficiency, Grant 614). The funders had no role in study design, data collection and analysis, decision to publish, or preparation of the manuscript.

## CONFLICT OF INTEREST STATEMENT

Susan Tuddenham receives royalties from UPTODATE and participates in research supported by in‐kind donation of test kits from Hologic. Jacques Ravel receives consulting fees from Biocodex where he serves as an International Board Member, manages US Patent 18/904823, is part of the Microbiome Committee (ISSVD, unpaid), and is the co‐founder of LUCA Biologics and a scientific adviser for Ancilia Bio. Michael T France has IP licensed (JR‐2020‐020 Using Lactobacillus Asparagine Synthase Protein Variant to Predict Colonization Stability and as Anti‐Gardnerella in Vaginal Microbiota Modulation Strategies), receives funding from the Bill and Melinda Gates Foundation to travel to the VMRC annual meeting, manages US Patents (issued US Patent 10 967 012—Microbiome‐based informed method to formulate live biotherapeutics; issued US Patent 11 037 655—Microbiome‐based informed method to formulate live biotherapeutics; issued US Patent 11 464 813—Microbiome‐based informed method to formulate live biotherapeutics; issued US Patent 11 389 491—Microbiome‐based informed method to formulate live biotherapeutics; pending WO2023168275—Vaginal live biotherapeutic compositions and methods of use thereof). All other authors have declared that no competing interests exist.

## ETHICS STATEMENT

The JiVitA‐1 study received ethical approval for all study procedures, including the verbal consent process, from the Johns Hopkins School of Public Health Institutional Review Board in Baltimore, Maryland, United States, on May 18, 2001, and the Bangladesh Medical Research Council in Dhaka, Bangladesh, on May 8, 2001. All participants provided verbal informed consent. JiVitA data collectors visited participants in their homes to obtain and document verbal consent for study participation. The trial is registered at clinicaltrials.gov (NCT00198822).

## Supporting information


Data S1.

## Data Availability

All relevant data are within the manuscript. Datasets used and/or analyzed during the current study are available from the corresponding author on reasonable request.
